# Sintilimab-Induced Myocarditis Overlapping Myositis in a Patient With Metastatic Thymoma: A Case Report

**DOI:** 10.3389/fcvm.2021.797009

**Published:** 2021-12-23

**Authors:** Zi-xuan Yang, Xuan Chen, Si-qi Tang, Qing Zhang

**Affiliations:** Department of Cardiology, West China Hospital, Sichuan University, Chengdu, China

**Keywords:** immune checkpoint inhibitor, sintilimab, myocarditis, myositis, myasthenia gravis

## Abstract

Although immune checkpoint inhibitor (ICI)-related myocarditis has been widely discussed, a lot of gaps and challenges in its clinical course and rational intervention remain elusive. We present the case of a 33-year-old man with a history of metastatic thymoma who developed dyspnea and muscle weakness 1 month after the first dose of sintilimab. He was asymptomatic but found to have a mild elevation of troponin-T and a moderate increase of creatine kinase 20 days after the infusion. Although the scheduled second dose was deferred, he developed dyspnea, left bundle branch block, and left ventricular enlargement that is suggestive of Grade 3 ICI-related myocarditis, complicated with myositis/myasthenia gravis 10 days later. Fortunately, his response to intensive immunosuppressive therapy was good.

## Introduction

Immune checkpoint inhibitor (ICI) has emerged as a revolutionized therapy across multiple refractory malignancies. Programmed cell death receptor 1 (PD-1) or programmed cell death ligand 1 (PD-L1) is among the most established targets for immunological tumor depletion. However, while T cells are inflamed against tumor cells, undesired withdrawal of tolerance to self-antigens has created a wide spectrum of immune-related adverse events (irAEs) (1). ICI-related myocarditis as one of the life-threatening irAEs has been broadly discussed in the previous publications (2), but the gap of knowledge remains in terms of its clinical pattern (3). Sintilimab, a human monoclonal antibody targeting PD-1, was recently approved in China and launched extensive clinical trials (4). In this case study, we reported a rare case of myocarditis overlapping myositis/myasthenia gravis (MG) induced by sintilimab with the description of an “incubation” before the onset of severe symptoms.

## Case Presentation

**Figure 1:**
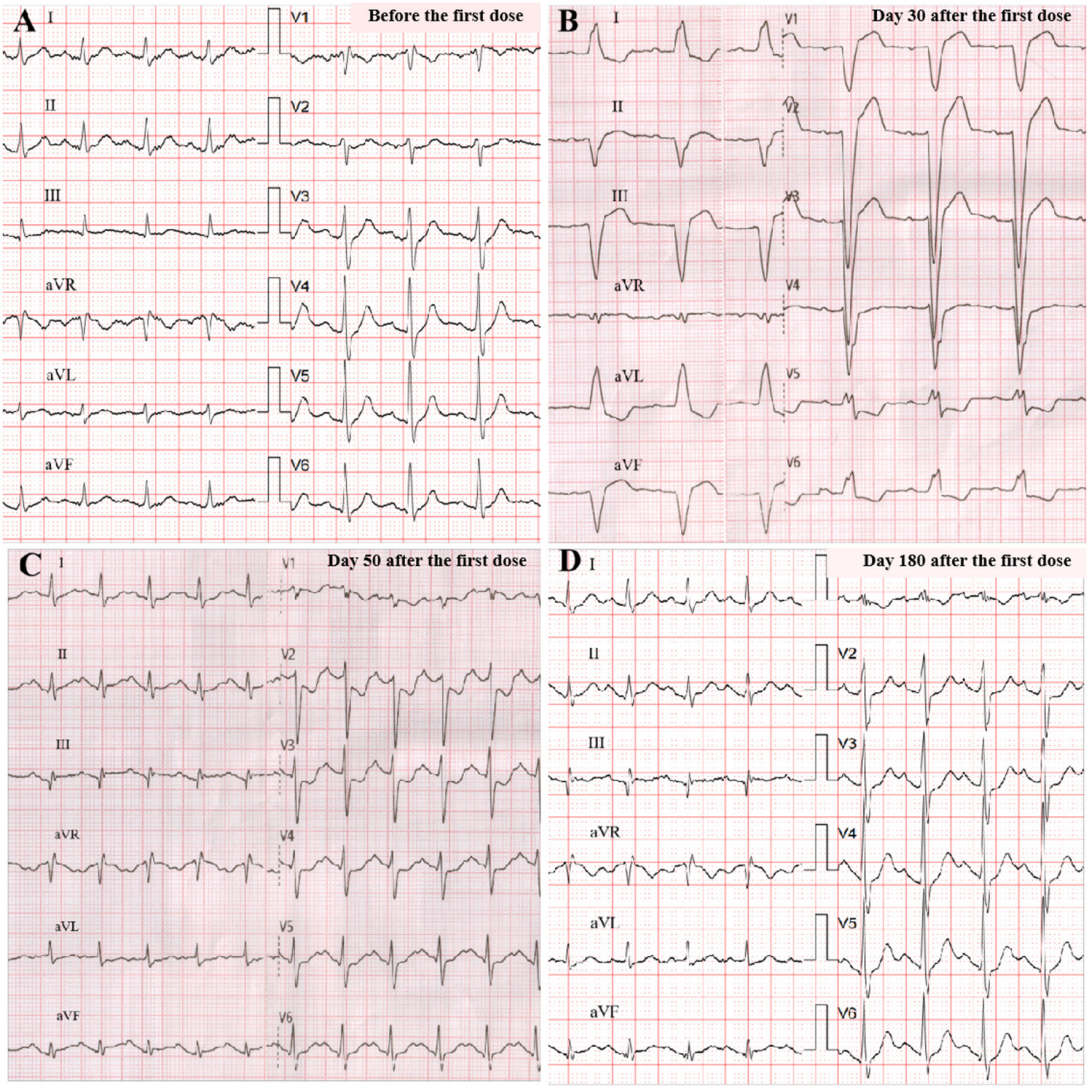
ECG changes. Before programmed cell death receptor 1 (PD-1) inhibitor treatment (sinus 104 bpm, normal QRS of 106 ms) **(A)**; on admission (sinus 73 bpm, wide QRS of 156 ms, left bundle branch block) **(B)**; at discharge (sinus 125 bpm, normal QRS of 118 ms) **(C)**; at 6-month follow-up (sinus 113 bpm, normal QRS of 110 ms) **(D)**.

**Figure 2:**
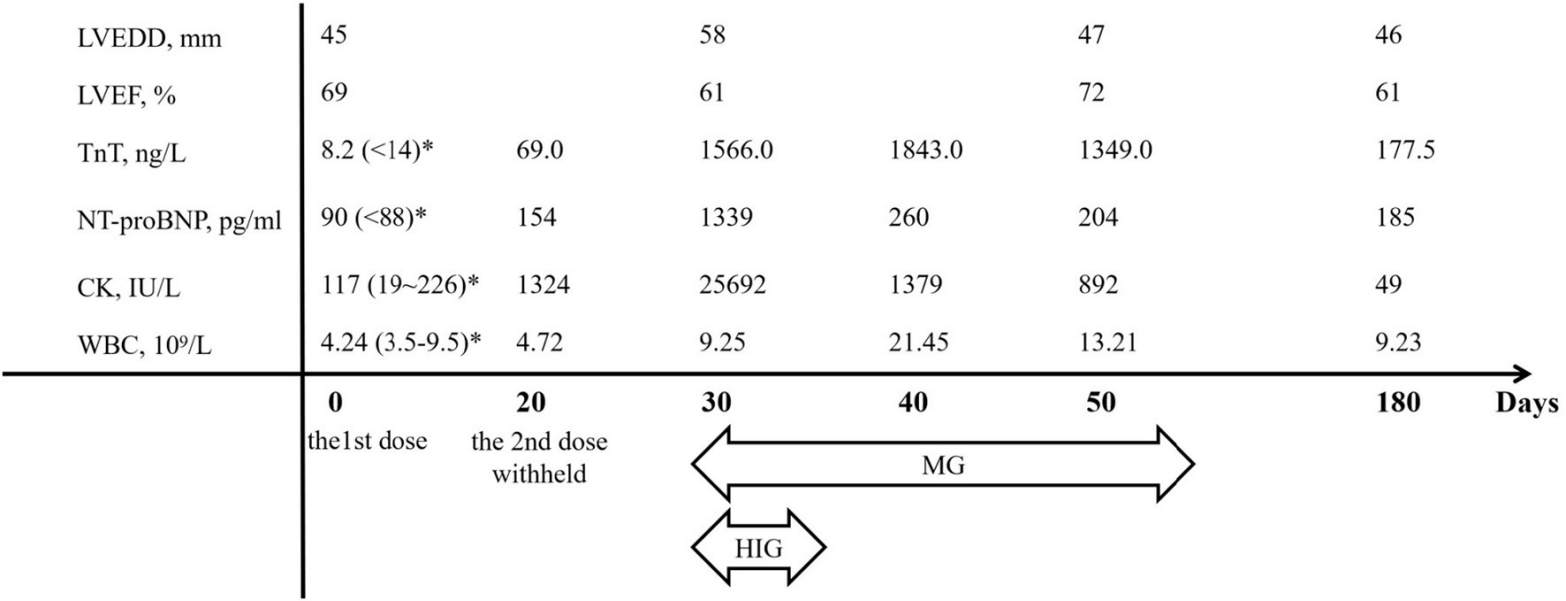
The chronological changes of major parameters. CK, creatine kinase; HIG, human immunoglobulin; LVEDD, left ventricular end-diastolic dimension; LVEF, left ventricular ejection fraction; MG, methylprednisolone; NT-proBNP, N-terminal pro-brain natriuretic peptide; TnT, troponin-T; WBC, white blood cell count. *Normal ranges in parentheses.

A 33-year-old male with a history of metastatic thymoma was admitted with dyspnea, palpitation, and muscle weakness 1 month after the first infusion of sintilimab. He had received multiple lines of therapy before a high expression (50%) of PD-L1 was detected by immunohistochemical examination. He had no history of hypertension, diabetes, dyslipidemia, smoking, or drinking. He was given the first dose of 200 mg sintilimab intravenously after a normal baseline assessment including cardiac biomarkers, ECG, and echocardiography. A total of 24 days later, proactive monitoring before the next dose revealed an elevated serum troponin-T (TnT) of 69 ng/ml (normal <14 ng/l), N-terminal probrain natriuretic peptide (NT-proBNP) of 154 ng/ml (normal <88 ng/ml), and creatine kinase (CK) of 1,324 IU/L (normal 19–226 IU/l), though the patient was asymptomatic with unremarkable ECG and echocardiography. The second dose of sintilimab was deferred; however, he quickly developed dyspnea, palpitation, and muscle weakness in 10 days. Physical examination on admission showed a normal body temperature of 36.5°C, a regular heart rhythm of 78 bpm, and a mildly high blood pressure of 140/90 mmHg. Ptosis and dysarthria were noted, but no edema in the lower limbs. Markedly increased TnT (1,566 ng/ml), NT-proBNP (1,339 ng/ml), or CK (25,692 IU/l) level was alarmed. ECG demonstrated a new complete left bundle branch block with a QRS duration of 156 ms. Echocardiography indicated a dilated left ventricle (LV) with an LV end-diastolic dimension of 58 mm, but a preserved ejection fraction of 61%. Therefore, the diagnosis of Grade 3 ICI-related myocarditis, MG was suggested, and a combination therapy of methylprednisolone (2 mg/kg/d), human immunoglobulin (20 g/d for 5 days), and pyridostigmine (180 mg/day) was given. Within a few days after the treatment, his symptoms significantly improved, while the LV reduced to a normal size and the QRS complexes resumed a normal morphology (Figures 1A–D). He maintained oral prednisone in a tapering regimen for 6 months. The chronological changes in biomarkers and LV parameters with immunosuppressive treatment are shown in Figure 2.

## Discussion

Few ICI-based therapies were available for patients with thymoma in China. Clinical trials reported that severe irAEs occurred in 15% of the patients, with pneumonitis being the most frequent (4). Previous publications with other ICIs suggested 25 and 11% of concomitant myositis and MG among myocarditis, respectively (5), which indicated common inciting autoantibodies. Patients with preexisting autoimmune disease (AD) are potentially more susceptible to irAEs. A phase 2 study of pembrolizumab observed a higher incidence of cardiac and muscular abnormalities in patients with thymic carcinoma and a notable predilection to Asians. (6) Two recently reported cases of sintilimab-induced myocarditis and myositis or MG demonstrated some similarities in clinical pattern with other ICIs (7, 8). Comparatively, the current case showed an early onset after the first dose, a preceding asymptomatic period, a dramatic change of ECG, and a better outcome in a younger patient. Given, the recognized association of thymoma to AD, it would be rational to be alerted when treating this cohort (9).

With CD8+ T cell infiltration on endomyocardial biopsy being the cardinal feature of ICI-related myocarditis, changes detected by other noninvasive tools, such as ECG, echocardiography, and cardiac magnetic resonance are heterogeneous and inconclusive (3). Despite not being indicative to specific etiology, elevated troponin is the most sensitive marker of myocardial damage (10). In this case, a slight elevation of high-sensitive TnT was noted in proactive monitoring before the scheduled second infusion (usually 14–21 days after the first dose), which may not necessarily suggest overt myocarditis in the clinical settings other than ICI induced. Nonetheless, it was followed by a 2-week silent “incubation” period before an abrupt change into acute symptomatic heart failure with demonstrated electrical and structural abnormalities in the LV. We address this course because the progression in asymptomatic patients with only mild changes of cardiac biomarkers is not fully delineated, which could be a transient alteration, or as in our case, a preceding sign of a more severe event. Current consensus recommends holding the ICI without additional prophylaxis for this cohort, yet the evidence is mostly empirical (11).

## Data Availability Statement

The original contributions presented in the study are included in the article/supplementary material, further inquiries can be directed to the corresponding author/s.

## Ethics Statement

Written informed consent was obtained from the individual(s) for the publication of any potentially identifiable images or data included in this article.

## Author Contributions

Z-xY: contributed to data analysis, writing of the article, and editing of the figure. XC: contributed to imaging analysis and editing of the figure. S-qT: contributed to the literature search and clinical record collection. QZ: contributed to the clinical care of the patient, study design, and critical review of the article. All authors contributed to the article and approved the submitted version.

## Conflict of Interest

The authors declare that the research was conducted in the absence of any commercial or financial relationships that could be construed as a potential conflict of interest.

## Publisher's Note

All claims expressed in this article are solely those of the authors and do not necessarily represent those of their affiliated organizations, or those of the publisher, the editors and the reviewers. Any product that may be evaluated in this article, or claim that may be made by its manufacturer, is not guaranteed or endorsed by the publisher.
